# Alzheimer’s amyloid-β A2T variant and its N-terminal peptides inhibit amyloid-β fibrillization and rescue the induced cytotoxicity

**DOI:** 10.1371/journal.pone.0174561

**Published:** 2017-03-31

**Authors:** Tien-Wei Lin, Chi-Fon Chang, Yu-Jen Chang, Yi-Hung Liao, Hui-Ming Yu, Yun-Ru Chen

**Affiliations:** 1 Institute of Microbiology and Immunology, National Yang-Ming University, Taipei, Taiwan; 2 Genomics Research Center, Academia Sinica, Taipei, Taiwan; USF Health Morsani College of Medicine, UNITED STATES

## Abstract

Alzheimer’s disease (AD) is the most common dementia affecting tens of million people worldwide. The primary neuropathological hallmark in AD is amyloid plaques composed of amyloid-β peptide (Aβ). Several familial mutations found in Aβ sequence result in early onset of AD. Previous studies showed that the mutations located at N-terminus of Aβ, such as the English (H6R) and Tottori (D7N) mutations, promote fibril formation and increase cytotoxicity. However, A2T mutant located at the very N-terminus of Aβ shows low-prevalence incidence of AD, whereas, another mutant A2V causes early onset of AD. To understand the molecular mechanism of the distinct effect and develop new potential therapeutic strategy, here, we examined the effect of full-length and N-terminal A2V/T variants to wild type (WT) Aβ40 by fibrillization assays and NMR studies. We found that full-length and N-terminal A2V accelerated WT fibrillization and induced large chemical shifts on the N-terminus of WT Aβ, whereas, full-length and N-terminal A2T retarded the fibrillization. We further examined the inhibition effect of various N-terminal fragments (NTFs) of A2T to WT Aβ. The A2T NTFs ranging from residue 1 to residue 7 to 10, but not 1 to 6 or shorter, are capable to retard WT Aβ fibrillization and rescue cytotoxicity. The results suggest that in the presence of full-length or specific N-terminal A2T can retard Aβ aggregation and the A2T NTFs can mitigate its toxicity. Our results provide a novel targeting site for future therapeutic development of AD.

## Introduction

Alzheimer’s disease (AD) is the most common form of dementia occurred in the elderly that causes huge social and economic impacts in the world. Dementia describes significant loss of certain mental functions such as memory, attention, and abstract thinking. Amyloid-β (Aβ) deposition is the main pathogenic hallmark of AD besides hyperphosphorylated tau in neurofibrillary tangles[1]. Currently, several Aβ and tau positron emission tomography (PET) imaging probes have been developed[2] and the level of Aβ42 and tau in cerebrospinal fluids (CSF) have been used as references for disease progression. The current biomarker model for AD demonstrated that amyloid biomarkers including CSF Aβ42 level and Aβ PET show abnormality prior to the other biomarkers such as CSF tau level, functional PET scans, and cognitive impairment[3]. Whether Aβ or tau is responsible for the onset of AD is still under debate[2]. In preclinical AD that occurs approximately 10 to 15 years earlier than cognition decline, it is considered that Aβ aggregation plays a necessary role to drive tau abnormality leading to neurodegeneration[4–8].

Aβ is a proteolyzed peptide product from a type I transmembrane protein, amyloid precursor protein (APP), after sequential cleavages of β- and γ-secretases. The cleavage results in two main isoforms, Aβ40 and Aβ42, comprising 40 and 42 amino acids, respectively[1]. Aβ42 sequence is DAEFR HDSGY EVHHQ KLVFF AEDVG SNKGA IIGLM VGGVV IA. The N-terminal Aβ is more hydrophilic containing several charged residues, whereas the C-terminal region is more hydrophobic. Although Aβ40 is more abundant than Aβ42 in human body fluids, Aβ42 aggregates faster and is more detrimental to neuronal survival. Currently, Aβ is still the most targeted substance in AD therapeutic development.

Aβ is an intrinsically disordered peptide containing mostly random coils[9]. Aβ aggregation is initiated by changing their secondary structure from random coils to β-sheets and it further assembles into oligomers, protofibrils, fibrils, and senile plaques[10]. The aggregation is considered a nucleation-dependent polymerization with the nucleation as the rate limiting step. It first forms a nucleus and gradually elongates to form mature fibrils containing in-registered cross-β structures[11–13]. In general, Aβ fibrils have a diameter of ~10 nm and can be longer than 1 μm in length[14]. The fibrillization process can be probed by classic amyloid dyes, such as thioflavin T (ThT) that chelates cross-β-stands in fibrils then emits fluorescence[15]. Several fibril studies have concluded an Aβ40 fibril model with a salt bridge between D23 and K28[16] and a bend/turn-like structure between residues 23–29 flanked by two β-strands by residues 10–22 and 30–40[16–18]. It was reported that Aβ42 fibrils are composed of three β-sheets in residues 12–18, 24–33, and 36–40 and the β-strands form parallel β-sheets[19]. Furthermore, Aβ oligomers are heterogeneous. Some oligomers were reported to contain both anti-parallel and parallel β-sheets[20]. The N-terminus of Aβ is considered a flexible region and is not included in most structural models of fibrils and oligomers.

Majority of AD patients are sporadic. There are only less than 10% patients carrying genetic mutations, known as familial AD (FAD) that causes early onset of the symptoms. FAD mutations in APP sequence may occur outside or inside of Aβ region. The mutations can lead to increase of total Aβ production, increase of Aβ42/Aβ40 ratio, alteration in biophysical properties and assembly states of Aβ[21]. The identified mutations inside Aβ region are mainly located at the N-terminus or in the middle region within residues 21 to 23. They are English (H6R)[22], Tottori (D7N)[22, 23], Taiwan (D7H)[24], Flemish (A21G)[25], Arctic (E22G)[26], Dutch (E22Q)[27, 28], Italian (E22K)[29], and Iowa (D23N)[30]. Recently, genetic variation found in normal people and patients with FAD has revealed an important role for the very N-terminus of Aβ for risk of AD[22, 31–33]. Genetic variation in alanine 2 in Aβ sequence, or alanine 673 in APP, were reported to have either protective[34] or adverse effect[32, 33] on AD. A2T mutation reduces β-secretase mediated APP cleavages, and it is capable of retarding Aβ fibrillization[35], whereas A2V shows an acceleration effect on Aβ fibrillization[10, 35]. Meanwhile, N-terminus of Aβ is known to coordinate with several metal ions including copper, zinc, and iron[36].

Since Aβ aggregation is highly implicated in AD, understanding its misfolding mechanism are essential to develop targeted treatment. Inhibitors preventing Aβ self-assembly are potential therapeutic agents for AD[37]. In fact, many anti-aggregation compounds are shown to reduce cytotoxicity *in vitro* and *in vivo*[38]. Small molecule inhibitors were reported to directly or indirectly alter the aggregation pathways. The inhibitors can be small chemical compounds directly binding to Aβ or metal ion chelators to block Aβ-metal interaction[39–41]. Direct binding of the compounds may change Aβ conformation or block Aβ association to prevent aggregation. Meanwhile, others focused on short peptide inhibitors that could be a short sequence related or unrelated to Aβ peptide[42, 43]. For example, several studies have designed β-sheet peptide breakers against Aβ β-strands[42–44] and the short fragments derived from Aβ42 C-terminus could assemble into Aβ oligomers and protect neuron against the cytotoxicity[45].

In the past, due to the difference of Aβ isoforms resided in the C-terminus, the C-terminus of Aβ have drawn more attention from researchers on amyloid formation[46]. The role of N-terminal Aβ is mainly discussed in metal ion chelation, whereas, its role on aggregation is still not fully understood. Although the N-terminal Aβ is flexible in both oligomer and fibril structure studies, weakly clustered N-terminus was reported by a relaxation NMR study[47]. Interestingly, the antibody targeting Aβ residues 3–7 has the most beneficial effect[48] showing an intriguing role of N-terminal Aβ. Hence, in this study, we initiate to characterize the differences among wild type (WT) and N-terminal mutants focusing on A2T and A2V of Aβ40 peptide. The species were examined alone and in mixtures by fibrillization kinetics, secondary structures, morphology, and cytotoxicity. Nevertheless, we found that N-terminal fragments (NTFs) of A2T are able to inhibit and rescue Aβ-induced cytotoxicity. Our results showed that N-terminal Aβ is a potential target for AD therapeutic development and A2T NTFs can serve as new peptide inhibitors for Aβ aggregation.

## Materials and methods

### Aβ preparation

Aβ40 peptides including WT, A2T, A2V, and A2V NTF(1–10) were synthesized by the peptide synthesis core in Genomics Research Center, Academia Sinica by solid-phase peptide synthesis. A2T NTFs containing amino acids 1-x were purchased from GM biolab. Aβ variants were first dissolved in hexafluoroisopropanol (HFIP) and lyophilized. The lyophilized full-length Aβ peptide powder was re-suspended in DMSO and added to 10 mM phosphate buffer, pH 7.4. The A2T and A2V NTFs were directly dissolved in 10 mM phosphate buffer, pH 7.4. The full-length protein concentration was re-quantified by absorbance at 280 nm with extinction coefficient[49], 1,280 M^-1^ cm^-1^ and NTFs protein concentration was measured by bicinchoninic acid assay due to lacking of aromatic residues.

### ThT assay

Aβ at 25 μM were prepared in 10 mM phosphate buffer, pH 7.4, and monitored by addition of 5 μM ThT in quiescence. ThT fluorescence intensity was examined at 485 nm where the excitation was at 442 nm in a microplate reader (SpectraMax M5; Molecule Devices) at 25°C, The signals were collected every hr automatically with 1 min mixing before the measurement. Finally, the signals were calculated and averaged from three repeats with buffer background subtraction. The signals were normalized to the final plateau of WT Aβ.

### Transmission electron microscopy (TEM)

The 400-mesh Formvar carbon-coated copper grids (EMS electron Microscopy Sciences, Hatfield, PA, USA) were first discharged, then ten μl of end-point Aβ aggregates were placed on the grids for 5 min and washed by inversely placing the grids on top of a drop of distilled deionized H_2_O, 100 μl. The washes were performed three times with three separate drops. Then, the grids were stained by 10 μl of 2% filtered uranyl acetate (UA) for 1 min, washed by ddH_2_O inversely for three times, and air-dried at room temperature overnight. UA is a commonly used negatively stained reagent for TEM studies. UA enhances the contrast by interaction with proteins. The final UA concentration in the samples was 1%. The samples were scanned by Hitachi H-7000 transmission electron microscope. The accelerating voltage at 75 kV was used. Several images were collected in each sample with different magnification to observe amyloid morphology.

### Native PAGE

Freshly prepared Aβ variants were dissolved in 3 mM NaOH and sonicated 1 min. After lyophilization, Aβ peptide powder was dissolved in 50 mM phosphate buffer, pH 7.2, and centrifuged at 21,000g at 4°C. Native page was prepared with stacking and running gels containing 4% and 15% acrylamide in 0.375 M Tris-HCl, pH 8.8, respectively. The samples were diluted by 2X sample buffer that contains 62.5 mM Tris-HCl, pH 6.8, 25% glycerol, and 1% bromophenol blue. Thirty μl samples were loaded to the well and the electrophoresis was run at 90 V. The gel was transferred to 0.45 μm PVDF membrane (Millpore) and subjected to western blotting following the standard protocol. The antibodies used for western blotting were mouse anti-Aβ, 1–16 antibody 6E10 (1:5000) (Covance) and anti-mouse IgG (HRP) (GeneTex).

### Analytical ultracentrifugation (AUC)

Freshly prepared Aβ variants were prepared based on native PAGE protocol. The concentration of Aβ samples was re-quantified and diluted to a final concentration of 50 μM. After preparation, the Aβ variants were immediately examined in a ProteomeLab XL-I centrifuge with an An-60Ti rotor (Beckman Coulter, Brea, CA, U.S.). Sedimentation velocity (SV) experiments was conducted following previous literature[50]. Briefly, four hundred μl of 50 μM Aβ variants and 450 μl of buffer were loaded in 12-mm aluminum double-sector centerpieces and centrifuged at 60,000 rpm for 15 h at 4°C. Ultraviolet absorption was adopted with the time interval of two-minute between each scanning. Moving boundaries were analyzed by SEDFIT software from National Institutes of Health (NIH, U.S.) and parameters were calculated by SEDNTERP software developed from University of New Hampshire, USA.

### Circular dichroism (CD)

Freshly prepared Aβ variants were dissolved 3 mM NaOH and sonicated 1 min. After lyophilization, Aβ peptide powder was dissolved in 10 mM phosphate buffer, pH 7.2, and centrifuged at 21,000g at 4°C. The supernatant was re-quantified. Then, Aβ variants were diluted to final concentration of 50 μM. All measurements were performed at room temperature, and 10 accumulations were averaged on a JASCO J-815 spectropolarimeter (Jasco Inc., MD, USA). Spectra were obtained from 190 to 250 nm with buffer background subtraction. The molar ellipticity was calculated and plotted.

### Fourier transform infrared (FTIR) spectroscopy

The end-point products of Aβ were centrifuged at 17,000 x g, 4°C and washed by ddH_2_O for two times. The pellets were re-suspended by 10 mM phosphate buffer, pH 7.4. Ten μl sample was placed on the sample cell. The spectra were detected by Nicolet^™^ 6700 FT-IR spectrometers from Thermo Electron Corporation. Spectra were recorded at a resolution of 4 cm^-1^ and accumulated 30 times at a wave number range from 900 to 4,000 cm^-1^. The signals were calculated and normalized with the individual peak in max intensity.

### Heteronuclear Single Quantum Coherence (HSQC) analysis

The ^15^N-labeled Aβ40 was expressed in the M9 minimal media containing ^15^NH_4_Cl and purified by two affinity columns and one RP-HPLC purification as previously described[51]. In brief, a stock solution was prepared by dissolving recombinant ^15^N-labeled Aβ40 in 8 M Tris-buffered guanidine hydrochloride (GdnHCl), pH 7.4, at a concentration of 10 mg/mL, followed by rapid dilution into 10 mM Tris, pH 7.4, containing 10% D_2_O (9:1, v/v). Monomeric ^15^N labeled Aβ was obtained from the supernatant after centrifuging the stock solution at 17,000 × *g* at 4°C for 10 min to precipitate the aggregates and subjected to NMR studies. Full-length unlabeled WT, A2T, and A2V variants were dissolved in 8 M Tris-buffered GdnHCl, pH 7.4, at 15 mg/mL. Pre-aggregates were removed after centrifuging at 21,000 × *g*, 4°C for 10 min. Peptide concentration was determined using BCA assay. HSQC spectra were acquired using 50 μM recombinant ^15^N-labeled Aβ40 in Bruker Avance 600 or 800 MHz NMR spectrometer equipped with 5 mm triple resonance cryoprobe and Z-gradient at 278 K. Amide ^15^N/^1^H resonance assignment was based on BRMB number 17796[52] and TOCSY-HSQC of the control sample (^15^N-labeled Aβ40 with two molar equivalents of unlabeled WT). NMR chemical shift perturbation is calculated using the equation, CSP = sqrt(δ_H_^2^+0.2*δ_N_^2^). The rate of reduction is normalized by assuming intensity of Gly38 peak remains constant and determined by: 1-Int(A2T or A2V variants)/Int(WT control).

### Cell viability and cytotoxicity assay

MTT assay was employed to examine cell viability. Twenty-five μM of the end-point products of Aβ were diluted by DMEM/F12 media with the final working concentration at 10 μM and treated to human neuroblastoma SH-SY5Y cell line. After 24 hr incubation, MTT solution was added, and the cells were incubated for an additional 3 hr. Cells were lysed by 100% DMSO. The absorbance was measured at a wavelength of 570 nm by an ELISA reader (SpectraMax M5; Molecule Devices). The signals were subtracted from the buffer control, averaged, and normalized. The statistical analysis was performed by one-way ANOVA and Tukey’s Post Hoc Test in SPSS program (IBM, Armonk, New York, USA). LDH assay was used to examine cytotoxicity. For cytotoxicity of the end-point products, the end-point products of Aβ samples were treated to human neuroblastoma SH-SY5Y in DMEM/F12 media (GIBCO, Invitrogen) to the final concentration at 10 μM for one day and subjected to LDH assay. The cells were lysed by 2% Triton X-100 to serve as a positive control for 100% cytotoxicity. The substrate signal was monitored in an ELISA plate reader (SpectraMax M5; Molecule Devices). The substrate fluorescence was monitored and the kinetics of substrate increase was averaged and normalized to the positive control. The statistical analysis was performed by one-way ANOVA and Tukey’s Post Hoc Test in SPSS program (IBM, Armonk, New York, USA).

## Results

### Aβ A2T variant retards but A2V variant accelerates fibrillization

**Fig 1:**
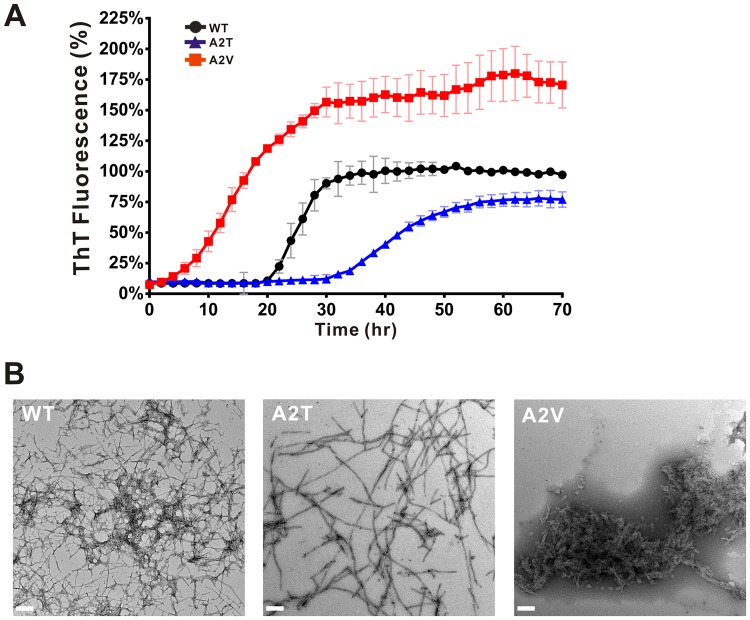
Fibrillization and fibril morphology of the Aβ variants. (A) Fibrillization kinetics of Aβ40 variants monitored by ThT assay in 10 mM phosphate buffer, pH 7.4. The peptide concentration was 25 μM. The signal was normalized to the plateau signal of WT. (B) TEM of the end-point products of Aβ variants. The scale bars are 200 nm.

To gain insight into the underlying mechanism of N-terminal Aβ variants focusing on residue 2, we first compared fibrillization of WT, A2T, and A2V Aβ40 by ThT assay (Fig 1A). We monitored aggregation of the three Aβ40 variants at 25 μM in 10 mM phosphate buffer, pH 7.4, with the presence of 5 μM ThT at 25°C. The results showed that WT fibrillized in a classic amyloid fibrillization pathway with a lag time of 20 hr and reached a plateau at 32 hr. Whereas, A2T extended the lag phase to about 30 hr and reached a plateau after 55 hr. The final ThT signal of A2T was approximately 25% lower in comparison to that in WT Aβ. In contrast, A2V rapidly fibrillized with a very short lag time and the signal reached a plateau at around 27 hr with over 60% more intensity than that of WT. The level of final ThT intensity indicates either the amount of fibrils formed in each variant or alteration of ThT fluorescence due to aggregate morphology. To confirm the formation of mature fibrils, we subjected the end-point products of each variant to TEM imaging (Fig 1B). The WT fibrils contains many crossed and branched fibers. These fibrils stacked in multiple bundles at the center of the clusters, whereas, at the outer region, single filaments were observed. A2T fibrils have much longer and less branched fibers. Little condensed region were found in A2T. In contrast, A2V fibrils are much clustered and dense. Many shorter and less branched filaments seem to pile into sheets and form large aggregates. Overall, TEM images showed that all variants formed fibrils in which A2V species contained much more clustered and dense fibrils in comparison to WT and A2T.

### Freshly prepared A2V monomer showed reduced random coil structures and A2T fibrils contain more α-helical structures

**Fig 2:**
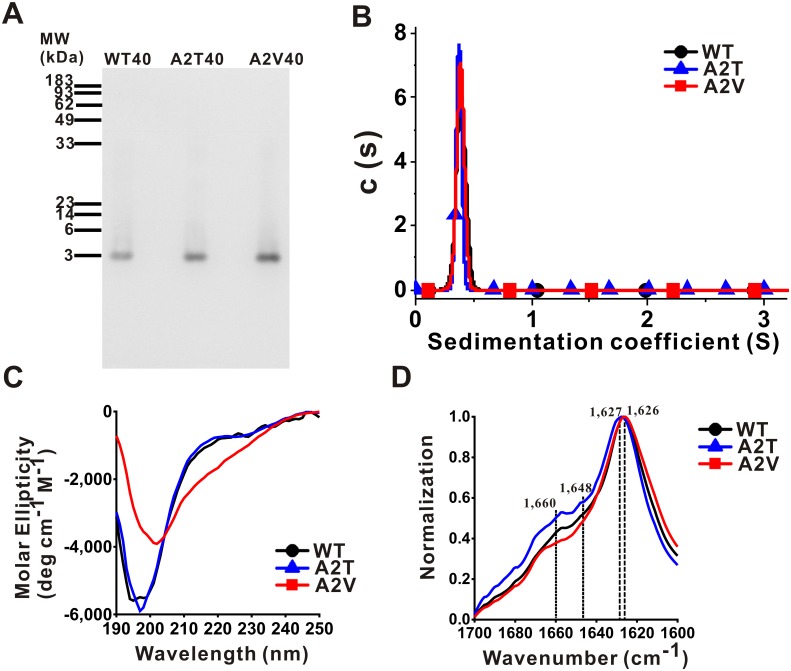
Native PAGE, AUC analysis, far-UV CD spectra, and FTIR spectra of the Aβ variants. (A) Native PAGE of freshly prepared WT, A2T, and A2V variants. (B) SV experiments of AUC of freshly prepared WT (black), A2T (blue), and A2V (red) variants. (C) Far-UV CD spectra of freshly prepared WT (black), A2T (blue), and A2V (red) variants. (D) FTIR spectra of fibrils of WT (black), A2T (blue), and A2V (red) variants. The peaks were indicated.

To gain insight into possible structural differences in freshly prepared and fibrillar Aβ variants, we first subjected the freshly prepared Aβ variants, i.e. WT, A2T, and A2V, to native PAGE (Fig 2A), SV experiments of AUC (Fig 2B), and far-UV CD spectroscopy (Fig 2C). Native PAGE and SV experiment were employed to analyze Aβ assembly. In native PAGE, all three freshly prepared Aβ variants migrated as single bands around 4 kDa. In SV studies, the data showed a single species at 0.389, 0.373, and 0.384 S for Aβ40WT, A2T, and A2V, respectively (Fig 2B). The calculated molecular mass were 4,329, 3,832, and 4,132 Da. The data match the actual molecular mass qualitatively for Aβ40WT, A2T, and A2V monomer, which are 4,330, 4,360, 4,358 Da, respectively. There is no significant peak observed above 1 S. Therefore, both native PAGE and SV result demonstrate that that all freshly prepared Aβ variants are monomeric. In far-UV CD studies, we found freshly prepared WT and A2T monomers have overlapped spectra showing the similarity of secondary structure between them. However, freshly prepared A2V monomer showed a significant reduction of random coil structures indicated by reduced signal around 200 nm and may have more β-content than WT and A2T as indicated by increased signal around 216 nm. Meanwhile, we collected the fibrils precipitated from the end-point products of aggregation, washed, and subjected them to FTIR spectroscopy. The fibrils were precipitated, washed, and re-suspended in 10 mM phosphate buffer, pH 7.4 as described in methods. FTIR spectra from 1,600 to 1,700 cm^-1^ were measured and normalized. The FTIR spectra showed great similarity among the three fibril samples. The secondary structures of the fibrils were all enriched in β-structures as evidenced by the peak near 1,620 to 1,640 cm^-1^[53]. The typical parallel β sheet structure was observed in wavenumber of 1,626 cm^-1^ for WT and A2V and 1,627 cm^-1^ for A2T fibrils. We found WT and A2V fibrils have similar spectra around 1,645 to 1,662 cm^-1^, a region indicates α-helical structures. A2T fibrils have ~13% more spectral intensity for α-helical structures in comparison to that of WT fibrils. In contrast, A2V fibrils do not contain obvious peaks in this region. The CD and FTIR results showed that mutation at residue 2 has the ability to change secondary structure content of freshly prepared and fibrillary Aβ variants.

### The full-length A2T and A2V variants and their N-terminal fragments (NTFs) interfere WT Aβ fibrillization

**Fig 3:**
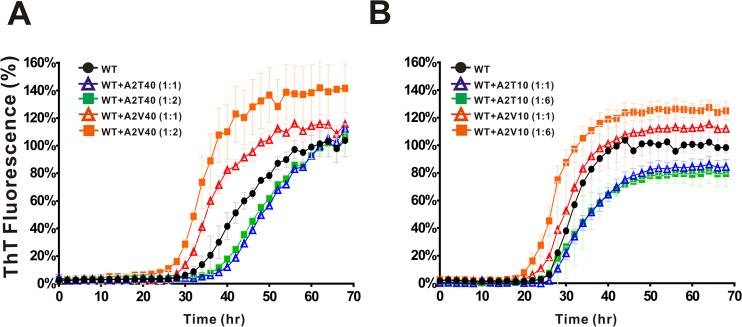
The effects of full-length and N-terminal A2T and A2V variants on Aβ fibrillization. Fibrillization kinetics of WT Aβ40 co-incubated with different ratios of (A) full-length of A2T or A2V and (B) NTF of A2T or A2V from residue 1 to 10. ThT assay was performed in 10 mM phosphate buffer, pH 7.4. WT Aβ40 was 25 μM. The WT to full-length A2T/A2V ratio were 1 to 1 and 1 to 2. The WT to A2T/A2V NTF ratio were 1 to 1 and 1 to 6. The signal was normalized to the plateau signal of WT.

Since heterozygous APP is appeared in people carrying the familial mutations, we examined the fibrillization of mixtures of WT and A2T or A2V (Fig 3A) to mimic the possible physiological condition. Freshly prepared WT Aβ40 at 25 μM was co-incubated with one and two fold of full-length A2T/A2V in quiescence and monitored fibril formation by ThT signal. We found A2V mutant promoted WT Aβ fibril formation in a dose-dependent manner by shortening lag time for 4 hr for 1 to 1 molar ratio and 6 hr for 1 to 2 molar ratios of WT to A2V. The ThT signal of WT mixed with two fold A2V in the final plateau increased about 40% than WT alone. Conversely, A2T retarded WT fibril formation and extended the lag phase for about 8 hr for both 1 to 1 molar ratio and 1 to 2 molar ratios of WT to A2T. We are curious about the possible effect attributed from the N-terminal region where the mutation resides. To examine whether the effect can be attributed from NTF, we used NTFs ranging from residues 1 to 10 of A2T and A2V to co-incubate with WT Aβ. The ratio of WT to A2T and A2V NTF was 1 to 1 and 1 to 6 (Fig 3B). Interestingly, we found A2V NTF(1–10) could still accelerate fibril formation. A2V NTF shortened the lag phase for around 4 hr for 1 to 1 molar ratio and around 8 hr for 1 to 6 molar ratios. ThT signal of the end-point product in the presence of 1 fold of A2V NTF increased ~10% and in 6 fold A2V NTF(1–10) increased ~22% in comparison to WT only. Furthermore, A2T NTF(1–10) are still capable of retarding fibril formation by slowing the elongation rate and decreasing the amount of fibrils for about 20% at the plateau. However, unlike full-length A2T the extension of the lag time by A2T NTF is not significant. The result demonstrated that NTFs are able to interfere with Aβ fibrillization.

### NMR spectra of WT Aβ40 showed large chemical shifts upon co-incubation with full-length A2V

**Fig 4:**
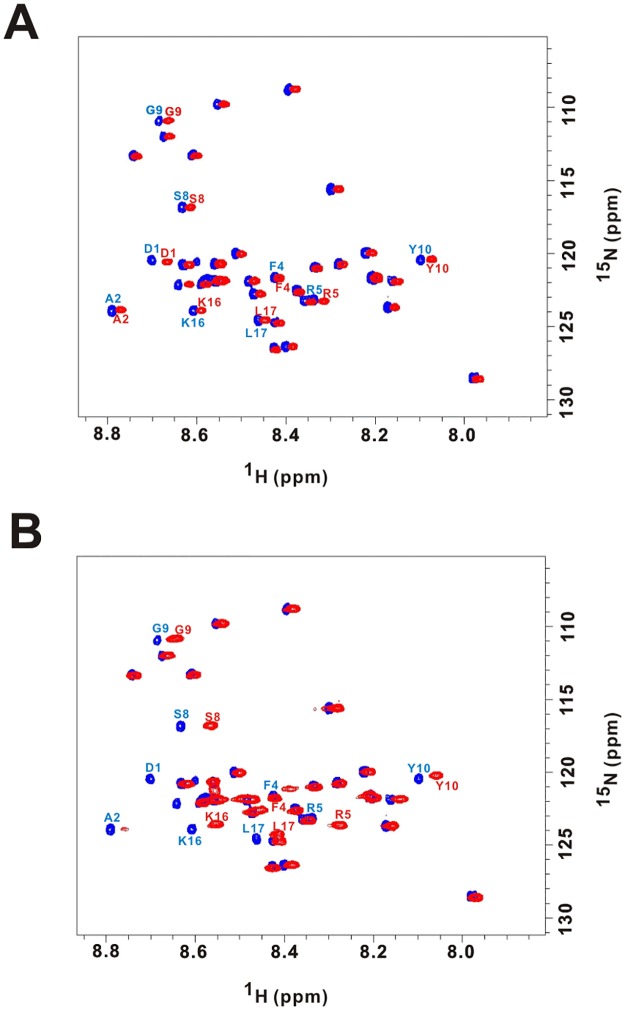
HSQC spectra of ^15^N labeled Aβ40 co-incubated with full-length WT, A2T, or A2V Aβ40. (A) ^15^N labeled Aβ40 co-incubated with two molar equivalents of full-length WT (blue) or A2T (red). (B) ^15^N labeled Aβ40 co-incubated with two molar equivalents of full-length WT (blue) or A2V (red).

**Fig 5:**
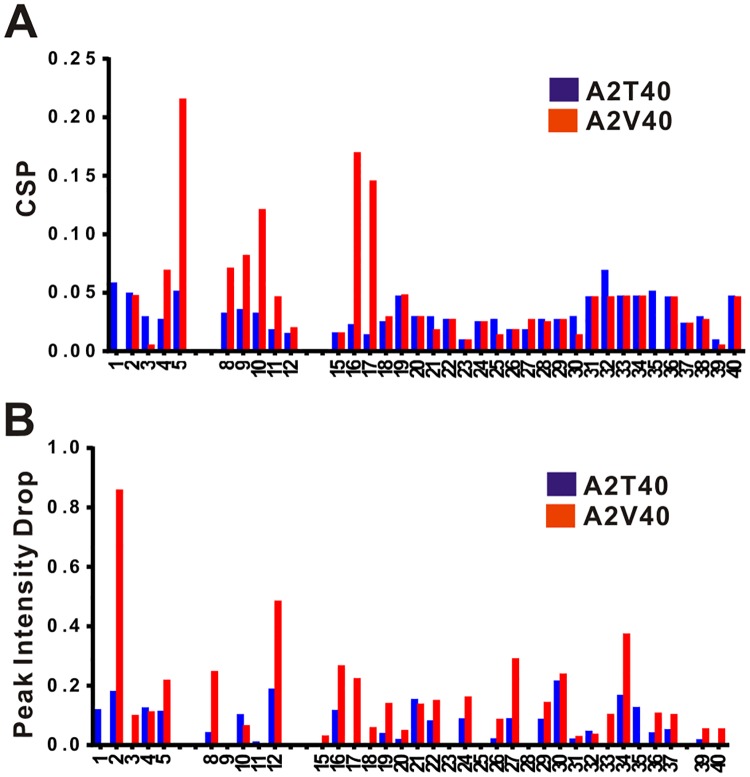
NMR CSP and cross peak intensity differences in Aβ40 co-incubated with full-length of WT (control), A2T, and A2V variants. (A) The CSPs between control and A2T (Blue) or A2V (Red). (B) The differential reduction of cross peak intensity between control and A2T (Blue) or A2V (Red).

To gain more insight into the effect of A2 mutation to the fibrillization of Aβ, we collected ^1^H-^15^N HSQC of ^15^N labeled Aβ40 at 50 μM by NMR spectroscopy upon titration with two molar equivalents of unlabeled WT (control), A2T, or A2V variants at 278 K. The total Aβ concentration was 150 μM. The spectra of full-length WT Aβ40 alone (blue cross peaks) and WT Aβ40 with 2 molar equivalent of full-length Aβ40 A2T (Fig 4A) and A2V (Fig 4B) (red cross peaks) are shown. The cross peaks were labeled with amino acid abbreviation and residue number according to our assignment. Comparing the chemical shifts, we found addition of A2V caused more significant chemical shifts than addition of A2T, especially the residues in the N-terminus of Aβ40, including F4, R5, S8, G9, Y10, and the middle region, including K16 and L17. The chemical shift perturbation (CSP) between control and addition of A2T/A2V variants clearly shows that A2T and A2V variants interact with Aβ40 (Fig 5A). We found that those residues experienced larger differences are located at the N-terminus, residues 4, 5, 8–10, 16–17 of Aβ40 after titration with A2V. Besides, significant decrease in the intensities of Aβ40 cross peaks was observed upon A2V titration compared to A2T (Fig 5B), which may be resulted from precipitation or aggregation of Aβ40 after interacting with A2V. The percentage of intensity decreasing for residues A2, V12, and L34 were over 40% and residue R5, S8, K16, N27, A30 were around 20–30%. We also collected ^1^H-^15^N HSQC spectra after several weeks under 278 K, the HSQC of A2T sample remained the same but not the A2V sample (data not shown). These NMR results also demonstrated that the presence of A2V accelerates fibril formation but A2T does not. The results are consistent with our fibrillization assays and the literature[10, 35].

### NTFs of A2T comprising resideus 1 to 7–10 could inhibit WT Aβ fibril formation and rescue Aβ induced cytotoxicity

**Fig 6:**
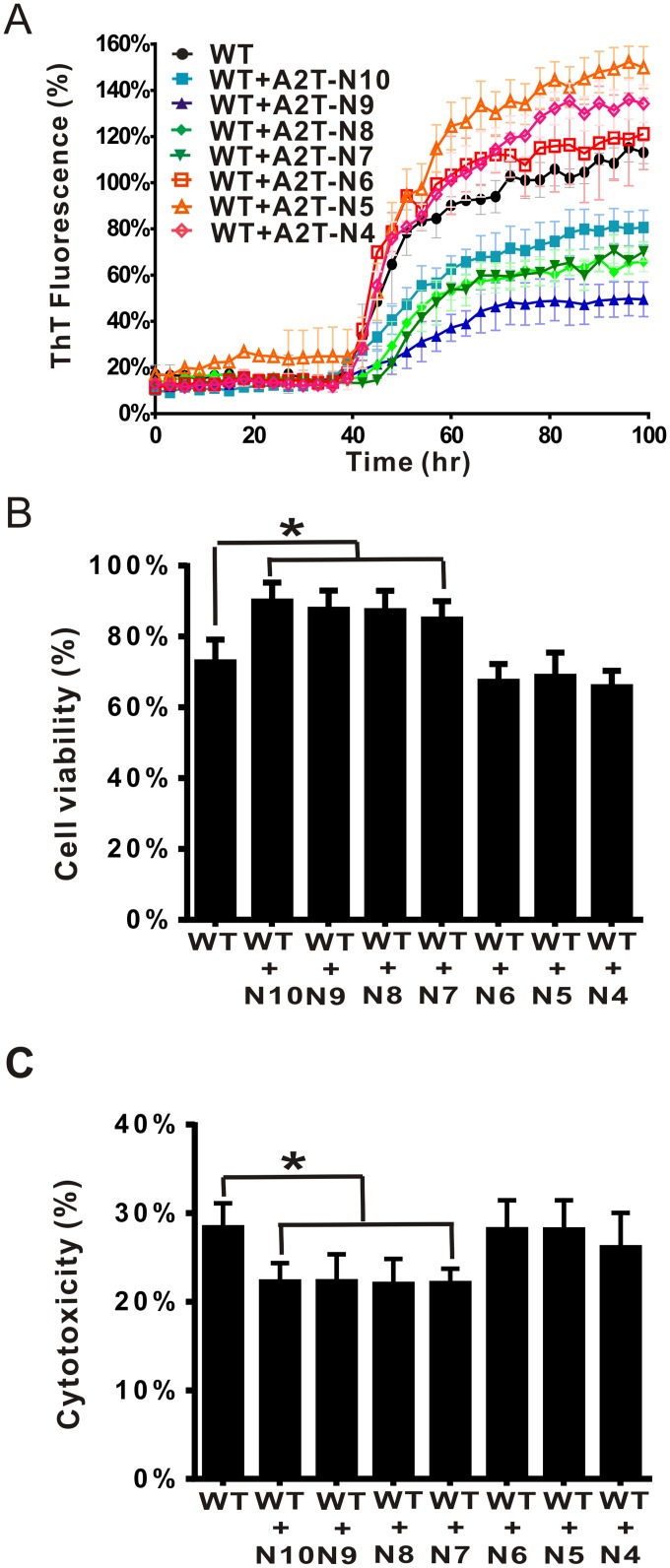
Fibrillization and cytotoxicity of WT Aβ40 co-incubated with A2T NTFs. (A) Fibrillization of Aβ40 in the absence and presence of A2T NTFs. Aβ40 WT co-incubated with A2T NTFs, monitored by ThT assay in 10 mM phosphate buffer, pH 7.4. Final Aβ concentration was 25 μM and A2T NTFs concentration were 125 μM. The signal was normalized to the plateau signal of WT. (B) Cell viability of Aβ in the absence and presence of A2T NTFs. The end-point products of ThT assay were treated to neuroblastoma SH-SY5Y cells with final concentration of 10 μM. After 1 day incubation, cell viability was measured by MTT assay. The data were normalized with buffer control. The effect of NTFs were compared with the cells treated WT Aβ alone using one-way ANOVA (*, p< 0.05). (C) Cytotoxicity of Aβ in the absence and presence of A2T NTFs. Cytotoxicity was examined by LDH assay. The end-point products of ThT assay were treated to neuroblastoma SH-SY5Y cells with final concentration of 10 μM. After 1 day incubation, cytotoxicity was measured by LDH assay. Triton X-100 was used as a positive control for 100% cytotoxicity.

**Fig 7:**
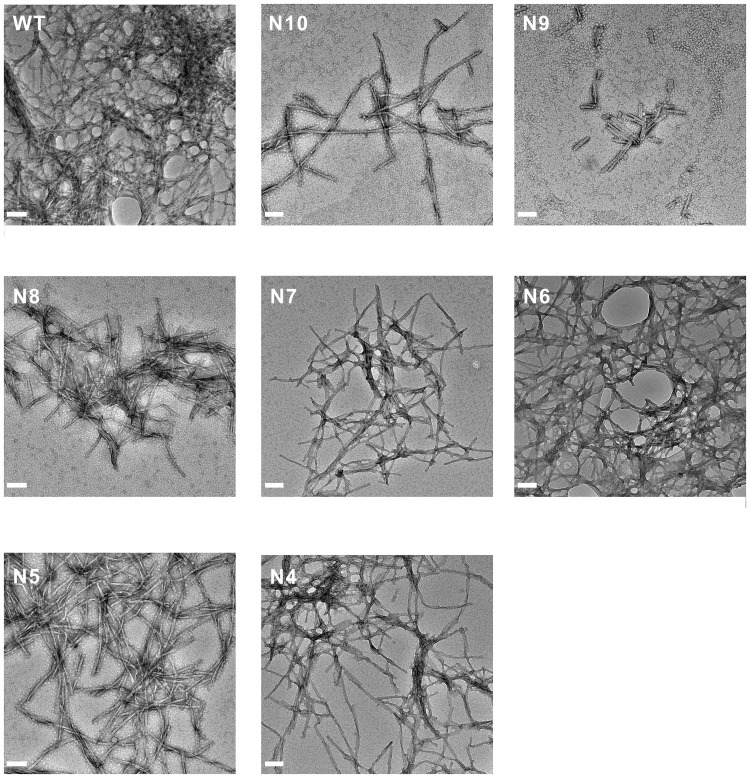
TEM images of the end-point products of Aβ incubated with various A2T NTFs. A2T NTFs ranged from residue 1-x, denoted as Nx (x = 4–10). The amino acid sequences are listed in Table 1. The TEM images of WT Aβ incubated with different A2T NTFs after 4 days in quiescence in 10 mM phosphate buffer, pH 7.4. Aβ40 WT concentration was 25 μM and A2T NTFs were 125 μM. The scale bars are 100 nm.

According to ThT assay, we found both full-length and NTF(1–10) of A2T have the capability to inhibit the fibril formation, we wonder if the effect can be also achieved by shorter NTF peptides. We used A2T NTFs ranging from residue 1 to residues 4 to 10, as listed in Table 1. Freshly prepared WT Aβ fibrillization at 25 μM was examined in ThT assay in the presence of different A2T NTFs at 5 molar equivalents. We found that only A2T NTF(1–10) to NTF(1–7) (denoted as A2T-N10 to A2T-N7) could decrease fibril formation, the percentage of decreased ThT intensity at the plateau was around 23–53% for N10, N9, N8, and N7. However, A2T NTF(1–6) to NTF(1–4) (denoted as A2T-N6 to A2T-N4) lost the inhibitive ability showing similar or even higher level of fibril formation to Aβ alone (Fig 6A). To examine whether the NTFs can increase cell viability and rescue the cytotoxicity induced by Aβ, we treated human neuroblastoma SH-SY5Y cells with the end-point products of the aggregation experiment and incubated for 24 hr at 37°C. Cell survival assay was performed by MTT assay (Fig 6B) and cytotoxicity assay was performed by LDH assay (Fig 6C). In MTT assay, Aβ alone treated cells has 72% cell survival comparing to buffer treated control (100% survival), that indicates Aβ alone causing ~28% toxicity. WT co-incubated with A2T NTF(1–6) to NTF(1–4) showed a similar level of cell survival. Whereas, WT co-incubated with A2T NTF(1–10), NTF(1–9), NTF(1–8) to NTF(1–7) were capable of significantly increase the cell survival to 84–90%. Furthermore, the cytotoxicity was performed by LDH assay. We found WT alone caused cytotoxicity for about ~27%, whereas A2T NTF(1–10), NTF(1–9), NTF(1–8) to NTF(1–7) were capable of significantly reducing Aβ-induced cytotoxicity to ~22%(Fig 6C). However, the effect only existed in longer NTFs but not in shorter NTFs (≤6 residues) in which the level of toxicity was still around 26–28%. The result indicates the need of residues 7 to 10 for the ameliorating effect. To further confirm the result and observe fibril morphology, we examined the end-point products by TEM imaging. In TEM, we observed fewer amount of fibrils in A2T NTF(1–10) to NTF(1–7) treatment, but not A2T NTF(1–6) to NTF(1–4) treatment (Fig 7). Our results demonstrated that NTFs comprising residues 1 to 7–10 of A2T are potential inhibitors for Aβ aggregation. In addition, to test whether A2T-NTFs can rescue the cytotoxicity induced by Aβ oligomers, Aβ40 derived diffusible ligands (ADDLs) according to the previous literature[54, 55] were prepared with and without the presence of A2T NTFs. The samples were treated to SH-SY5Y cells for LDH assay (S1 Fig). The result showed that WT ADDL comprising Aβ40 caused cytotoxicity about 13%, while WT in the presence of A2T NTF(1–10), NTF(1–9), NTF(1–8), and NTF(1–7) could significantly rescue ADDL-induced cytotoxicity (S1 Fig) to 10.6%. However, WT in the presence of A2T NTF(1–6), A2T NTF(1–5), and A2T NTF(1–4) did not rescue the cytotoxicity, ~14%. The results demonstrated that the longer A2T-NTFs are also able to rescue cytotoxicity induced by ADDLs.

**Table 1:** Amino acid Sequence of N-terminal A2T peptides.

NTF	Denotation	Sequence	Molecular weight (Da)
A2T(1–10)	N10	D**T**EFRHDSYG	1,226
A2T(1–9)	N9	D**T**EFRHDSY	1,063
A2T(1–8)	N8	D**T**EFRHDS	1,006
A2T(1–7)	N7	D**T**EFRHD	919
A2T(1–6)	N6	D**T**EFRH	804
A2T(1–5)	N5	D**T**EFR	667
A2T(1–4)	N4	D**T**EF	510

## Discussion

Clinically, A2T and A2V that mutate at the same site but different amino acids show either protective or exacerbating effect on AD. It is intriguing why changing amino acids at the same site would cause such an opposite effect even though changing alanine to valine increases hydrophobicity and to threonine increases polarity. Here, in this study we first characterized the properties of full-length A2T and A2V and the effects of full-length A2T and A2V in mixtures with WT. Our data are consistent with previous literature[35] showing A2V fibrillizes much faster and A2T retards fibrillization by extending lag phase. This effect may be attributed from conformational and assembly changes in monomer and oligomer stage. Indeed, previously ion mobility-mass spectrometry study showed that A2V/T influences on the oligomer assembly[56]. We further showed that A2V contains reduced random coil structures in comparison to WT and A2T in their monomer state. This result echoes the finding from molecular dynamic simulation that A2V Aβ42 has more populated double-hairpin structure in comparison to WT[57]. Also, we found the mixture of A2V/T with WT either accelerates or retards WT fibrillization in a dose-dependent manner and this effect can be observed when A2V/T NTFs were mixed with full-length Aβ. Our NMR studies revealed that A2V and A2T mainly influence the N-terminal and middle region of Aβ, where A2V induces aggregation. Consistently, in MD simulation form previous literature on Aβ42 they also found A2V promotes hydrophobic interaction between N-terminus region and central hydrophobic core to promote aggregation while A2T N-terminus forms electrostatic interaction between K16 and E22[57]. Furthermore, we discovered that the first 10 residues of NTFs of A2T and A2V are able to attribute to the opposite effect on Aβ fibrillization. We found for the first time that A2T NTFs ranging from residue 1 to 7 to 10, but not 1 to 6 or shorter, are capable to retard WT Aβ fibrillization and rescue Aβ-induced cytotoxicity. Our result demonstrated that changing interaction at the N-terminal regions is sufficient to alter Aβ fibrillization kinetics. This result may also explain the N-terminal Aβ targeting antibody has the most beneficial effect in previous literature[48].

It is interesting to discover the effect on N-terminal Aβ even though it is not considered in most of the oligomer and fibril models. However, Some Aβ40 fibril studies also demonstrated N-terminal residues 4–7 are involved in fibril structure[58–60], in which the Osaka mutant fibrils showed an intermolecular contact between residue 3 and residues 28–30. Most interestingly, Tycko’s group used solid state NMR to examine Aβ fibrils generated by seeding synthetic Aβ with brain extract of an AD patient and found F4, R5, D7, S8 contact intermolecular V24 and S26 of Aβ. The fibrils form 3-fold symmetry along the fibril growth axis. From H/D exchanged study, they suggest that the N-terminal segment indicated by A2, F4, D7, S8, G9, transiently unfolds and also exposes G25 and S26[60]. Besides, recently Aβ42 peptide in aqueous solution was identified with residual β-strand structures in 4 segments, residues 2–7, 16–23, 28–32, and 34–36[61]. In molecular dynamic simulation study of A2T, they found N-terminal of A2T forms unusual electrostatic interaction with K16 and E22 [57]. N-terminal mutation, modification, and/or truncation of Aβ have also been shown to alter fibrillization[62, 63]. For example, N-terminal truncated Aβ8–40 accelerates the aggregation kinetics compared with full-length Aβ1–40[62], N-terminal mutation at H6R or D7N accelerates fibrillization, and metal ions chelating N-terminal Aβ change the fibrillization pathway[31, 64–70]. Therefore, it is likely that N-terminal residues of Aβ has an impact on monomeric Aβ conformation and on the pathway to mature fibrils.

Peptide inhibitors have been a great interest for AD therapeutic development. The current reported peptides are mostly designed to break β-sheets[42, 43]. Several designs were based on Aβ sequence. For example, Aβ16–20 KLVFF derived β-sheet breakers including cholyl-LVFFA, methylated β-sheet breakers. Different lengths of C-terminal fragments (CTFs) of Aβ were also reported to be inhibitors to interfere with oligomer assembly[45]. They found Aβ(31–42) and Aβ(39–42) could inhibit intermolecular interactions and reduce cell toxicity. However, the CTFs ranging from residues 29–42 of Aβ are composed of mostly hydrophobic residues and the solubility is poor. CTFs may also potentially aggregate by themselves since the region is involved in both oligomer and fibril models. In our study, we found Aβ A2T(1–10) to (1–7) could inhibit fibril formation and rescue Aβ cytotoxicity. Since our NMR studies revealed that A2T influences the N-terminal and middle region of WT Aβ (Fig 5) and solid state NMR studies of authentic Aβ fibrils showed intermolecular contact of N-terminus and the middle region, V24 and S26, of Aβ[60], the potential mechanism of the inhibition may reside in interfering the WT Aβ assembly through N-terminal or middle region by A2T and A2T NTFs. The effective inhibition provided by A2T(1–7) but not (1–6) may indicate an important contact of D7[60]. Previously, D7 has been shown to contact with S26 in the Aβ fibril models[60], where S26 is in the important hinged region connecting the two intramolecular β-strands. A2T mutation may impact on D7 and make it more accessible to form such contact. Further molecular mechanisms related to A2T need to be investigated. The NTFs are highly soluble and do not form amyloids by themselves. Thus, NTFs of Aβ have good potential for therapeutic development as peptide inhibitors against Aβ aggregation in AD.

## Supporting information

S1 Fig Cytotoxicity of ADDLs in the absence and presence of A2T NTFs. Cytotoxicity was examined by LDH assay. The ADDLs of aggregation experiment were treated to neuroblastoma SH-SY5Y cells with final concentration of 25 μM. After 1 day incubation, cytotoxicity was measured by LDH assay. Triton X-100 was used as a positive control for 100% cytotoxicity. The statistical analysis was performed by one-way ANOVA and Tukey’s Post Hoc Test.
(TIF)

S1 File Method for ADDL oligomers cytotoxicity. (PDF)
