# Supplementary figures and images for: Alzheimer’s amyloid-β A2T variant and its N-terminal peptides inhibit amyloid-β fibrillization and rescue the induced cytotoxicity
Source: PLoS One. 2017 Mar 31;12(3):e0174561. doi: 10.1371/journal.pone.0174561 (PMC5376091; doi:10.1371/journal.pone.0174561)

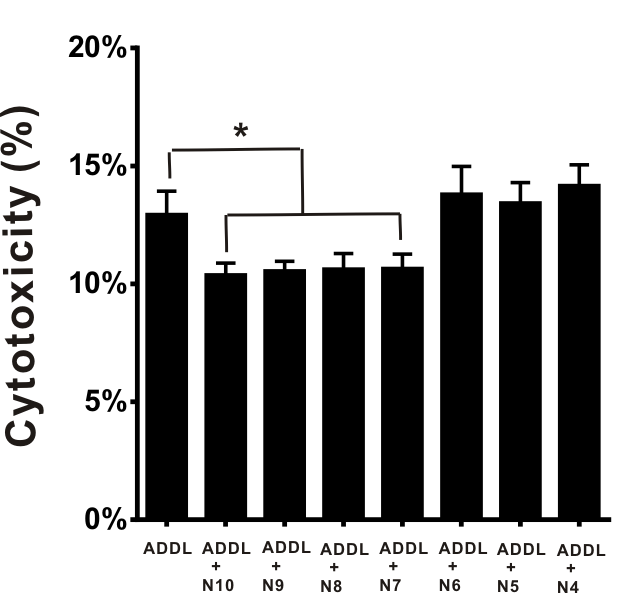

Supplement: S1 Fig — Cytotoxicity was examined by LDH assay. The ADDLs of aggregation experiment were treated to neuroblastoma SH-SY5Y cells with final concentration of 25 μM. After 1 day incubation, cytotoxicity was measured by LDH assay. Triton X-100 was used as a positive control for 100% cytotoxicity. The statistical analysis was performed by one-way ANOVA and Tukey’s Post Hoc Test. (TIF) [file pone.0174561.s001.tif]
