# Supplementary material for: Alzheimer’s amyloid-β A2T variant and its N-terminal peptides inhibit amyloid-β fibrillization and rescue the induced cytotoxicity
Source: PLoS One. 2017 Mar 31;12(3):e0174561. doi: 10.1371/journal.pone.0174561 (PMC5376091; doi:10.1371/journal.pone.0174561)
Supplement: S1 File — (PDF) [file pone.0174561.s002.pdf]

Supplemental Material and Method

ADDL oligomers cytotoxicity. Aβ40 WT peptide was re-suspended in HFIP and lyophilized. The lyophilized peptide powder was dissolved in DMSO at 5 mM and sonicated for 5 min. Then peptide solution was added to DMEM/F12 (GIBCO, Invitrogen) to stack concentration of 100 µM. ADDL and A2T-NTFs mixtures were prepared by mixing 25 µM ADDLs and A2T-NTFs 250 µM. The samples were incubated overnight at 4 °C. ADDLs on cytotoxicity were assessed using LDH assay. Human neuroblastoma SH-SY5Y cell line was treated with the ADDLs on aggregation experiment and incubated for 24 hr. Following the incubation, cells were lysed by 2% Triton X-100 to serve as a positive control for 100% cytotoxicity. The substrate signal was monitored in an ELISA plate reader (SpectraMax M5; Molecule Devices). The substrate fluorescence was monitored and the kinetics of substrate increase was averaged and normalized to the positive control. The statistical analysis was performed by one-way ANOVA and Tukey's Post Hoc Test in SPSS program (IBM, Armonk, New York, USA).
